# Simulated microgravity attenuates myogenesis and contractile function of 3D engineered skeletal muscle tissues

**DOI:** 10.1038/s41526-024-00353-z

**Published:** 2024-02-16

**Authors:** Zhanping Ren, Eun Hyun Ahn, Minjae Do, Devin B. Mair, Amir Monemianesfahani, Peter H. U. Lee, Deok-Ho Kim

**Affiliations:** 1 Department of Mechanical Engineering, Johns Hopkins University, Baltimore, MD 21205 USA; 2 Department of Biomedical Engineering, Johns Hopkins University, Baltimore, MD 21205 USA; 3 Department of Cardiothoracic Surgery, Southcoast Health, Fall River, MA 02720 USA; 4 Department of Pathology and Laboratory Medicine, Brown University, Providence, RI 02912 USA; 5 Department of Medicine, Johns Hopkins University, Baltimore, MD 21205 USA; 6 Center for Microphysiological Systems, Johns Hopkins University, Baltimore, MD 21205 USA

**Keywords:** Lab-on-a-chip, Diseases

## Abstract

While the effects of microgravity on inducing skeletal muscle atrophy have been extensively studied, the impacts of microgravity on myogenesis and its mechanisms remain unclear. In this study, we developed a microphysiological system of engineered muscle tissue (EMT) fabricated using a collagen / Matrigel composite hydrogel and murine skeletal myoblasts. This 3D EMT model allows non-invasive quantitative assessment of contractile function. After applying a 7-day differentiation protocol to induce myotube formation, the EMTs clearly exhibited sarcomerogenesis, myofilament formation, and synchronous twitch and tetanic contractions with electrical stimuli. Using this 3D EMT system, we investigated the effects of simulated microgravity at 10^−3 ^G on myogenesis and contractile function utilizing a random positioning machine. EMTs cultured for 5 days in simulated microgravity exhibited significantly reduced contractile forces, myofiber size, and differential expression of muscle contractile, myogenesis regulatory, and mitochondrial biogenesis-related proteins. These results indicate simulated microgravity attenuates myogenesis, resulting in impaired muscle function.

## Introduction

Mechanical cues such as stress and strain, shear flow, matrix stiffness, and topography alter gene expression and cell function^1–3^. Through a variety of mechanisms, including mechanical unloading and modification of systemic factors such as growth hormones, antioxidants, and vitamins^4^, microgravity can lead to loss of muscle mass and strength, also known as muscle atrophy^5–7^. Studies have shown long-term spaceflight results in reduced number and size of myofibers, protein synthesis rates, altered expression of metabolism-related genes, decreased myogenesis, and compromised mitochondrial function^7–11^. Genes involved in the regulation of mitochondrial activity have been shown to be differentially expressed in space, and these changes may play a key role in muscle loss and metabolic dysfunction both in vitro and in vivo^12,13^. Due to these modifications and their potential negative impacts on human health, skeletal muscle mechanisms involved in sensing and responding to changes in gravity have been extensively studied^4^. However, the expense of spaceflight research and the limited access to spaceflight platforms significantly restrict these research efforts.

The development of engineered muscle tissues (EMT) in the 1970s^14^ opened an era in myopathy research using an efficient and less costly tissue-engineered model. Polydimethylsiloxane (PDMS)-based tissue culture platforms were developed on which the muscle tissue is fabricated between two posts^15–18^. When electrically stimulated, EMTs generate contractile forces that subsequently cause deformation of the elastic PDMS post to which they are attached. Contractile forces can then be calculated based on PDMS mechanical properties using the Euler-Bernoulli beam bending theory. This allows for non-invasive and quantitative measurements of tissue physiological function. EMTs bridge the gap between 2D culture systems, animal models, and human data in terms of biological complexity, ease of use, experimental feasibility, and physiological relevance. As such, they have been used for biological research on the International Space Station (ISS) to study the impacts of spaceflight on muscle function. After spaceflight, EMTs exhibited the direct effect of microgravity at a tissue level, namely reduced protein synthesis rates and myofiber size, similar to what has been shown in astronaut and animal studies^19^. Such studies, however, have not assessed contractile performance.

The development of ground-based microgravity simulators, such as the random positioning machine (RPM), has provided a less costly and more readily available platform for conducting microgravity-related research on Earth^20^. By continuous programmed rotation at changing speeds and directions, the RPM theoretically cancels out the effects of unidirectional gravity over time, thus creating a simulated microgravity environment. Multiple studies showed that when exposed to simulated microgravity, myoblasts in monolayers exhibit reduced myogenic differentiation and reduced myotube size^21,22^. These studies, however, only investigated the effects of simulated microgravity myogenesis in 2D, which fails to accurately mimic in vivo cell-cell and cell-extracellular matrix (ECM) interactions.

Recently, researchers have advocated for strategies that leverage microgravity to promote biomanufacturing for regenerative medicine applications^23,24^. Despite this interest in biomanufacturing in space, the impacts of microgravity on 3D myogenesis remain poorly understood. In this study, we utilized our EMT platform to study the impacts of simulated microgravity on myotube formation from C2C12 murine myoblasts in 3D collagen / Matrigel-based hydrogels. Myogenesis in simulated microgravity and standard gravity were compared in terms of contractile force, myofiber morphology, and myofiber-specific protein expression. Our results demonstrate that 5-day simulated microgravity exposure impairs myogenic differentiation of myoblasts, leading to attenuated contractile performance, compromised structural characteristics, and reduced expression of specific structural proteins and mitochondrial regulators. This study represents an approach to studying skeletal muscle myogenesis in a microgravity environment using engineered skeletal muscle chips. Our miniaturized EMT platform enables non-invasive, real-time assessment of contractile functions, providing an in vitro model as an alternative to animal models to study physiological processes in space.

## Results

### Engineered skeletal muscles form multinucleated myotubular structures and exhibit contractile function

**Fig. 1:**
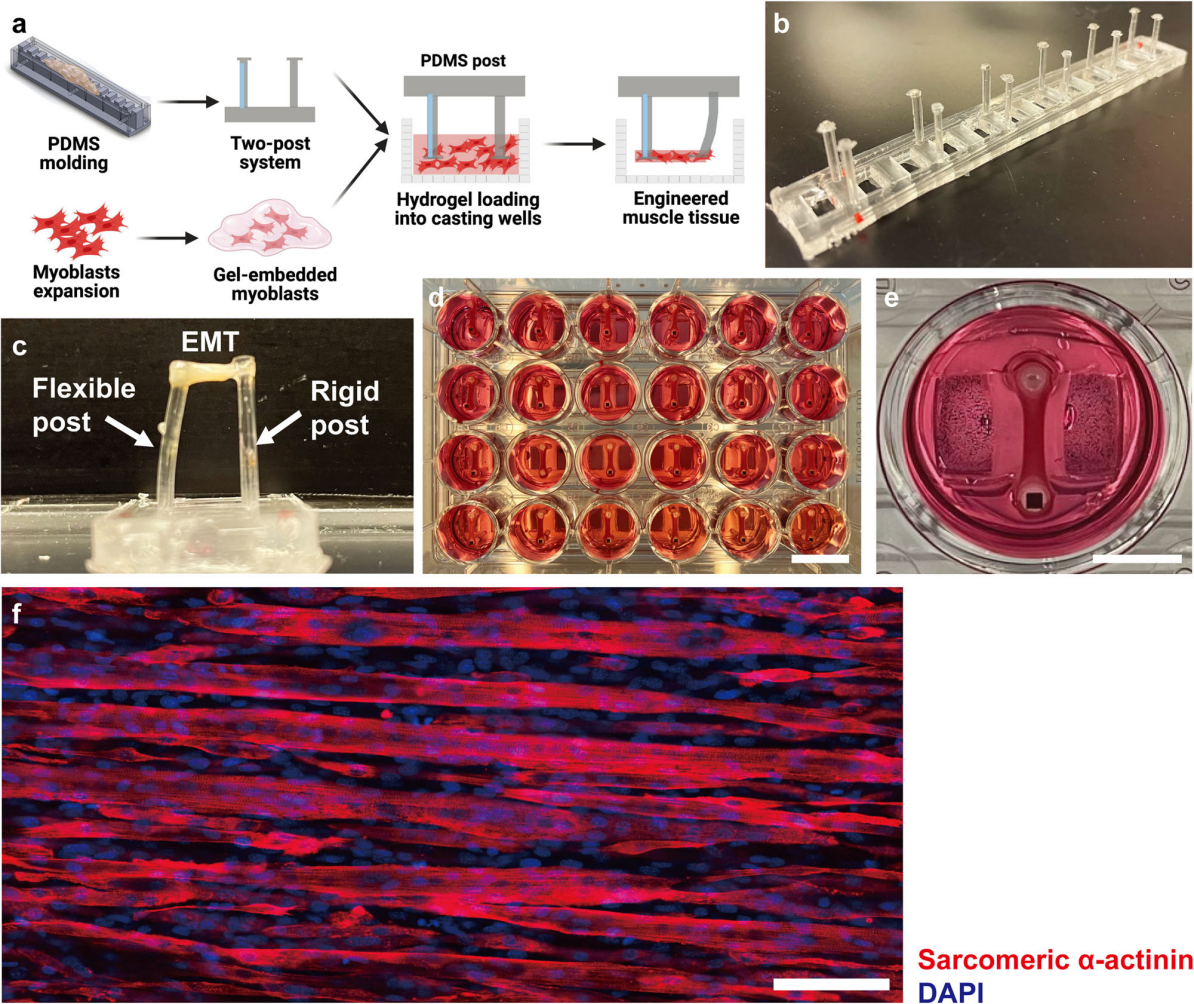
Engineered muscle tissues (EMTs) are fabricated using the PDMS two-post system and develop multinucleated myotubular structures in 5 days. **a** A schematic showing the EMT fabrication process based on collagen / Matrigel composite hydrogels that self-assemble around a PDMS post array. Created using Biorender^®^. **b** A PDMS post array consists of 6 post pairs, each with one flexible and one rigid post. **c** A representation of an EMT formed around the tips of a PDMS post pair. EMT contractile force and kinetics are determined from the displacement of the flexible post using Euler-Bernoulli beam theory. **d** A representative photograph of EMTs cultured in a full 24-well plate. Scale bar: 15 mm. **e** An EMT in an individual well of a 24-well plate. Scale bar: 5 mm. **f** Immunofluorescence staining for sarcomeric α-actinin and DAPI shows differentiated, multi-nucleated myotubes in a 5-day EMT whole mount. Scale bar: 50 μm.

We assessed development of physiological myotubular structure and contractile function in our EMTs to verify that this platform was applicable for studying the myogenic process in vitro. The EMT fabrication procedure is shown in Fig. 1a. PDMS post arrays consisting of rigid and flexible posts were fabricated using customized acrylic molds (Fig. 1b, Supplementary Fig. 1a). In the first 24 h following tissue fabrication, cell-loaded hydrogels solidified and started to compact into a dumbbell shape on the tips of PDMS posts (Fig. 1c–e), indicating focal adhesion mediated increases in physical interaction between cells and the ECM. Spontaneous contraction was observed in some EMTs as early as 3 days after switching to differentiation media (DM). Electrically induced muscle contraction has long been utilized to evaluate the excitability and functional properties of skeletal muscles^18,25,26^. Electrical stimulation of EMTs resulted in robust and synchronous contractions. After 5 days of culture in DM, EMTs exhibited further compaction, indicating elevated cell-matrix interaction and intercellular tension caused by formation of highly aligned and multinucleated myotubes (Fig. 1f).

**Fig. 2:**
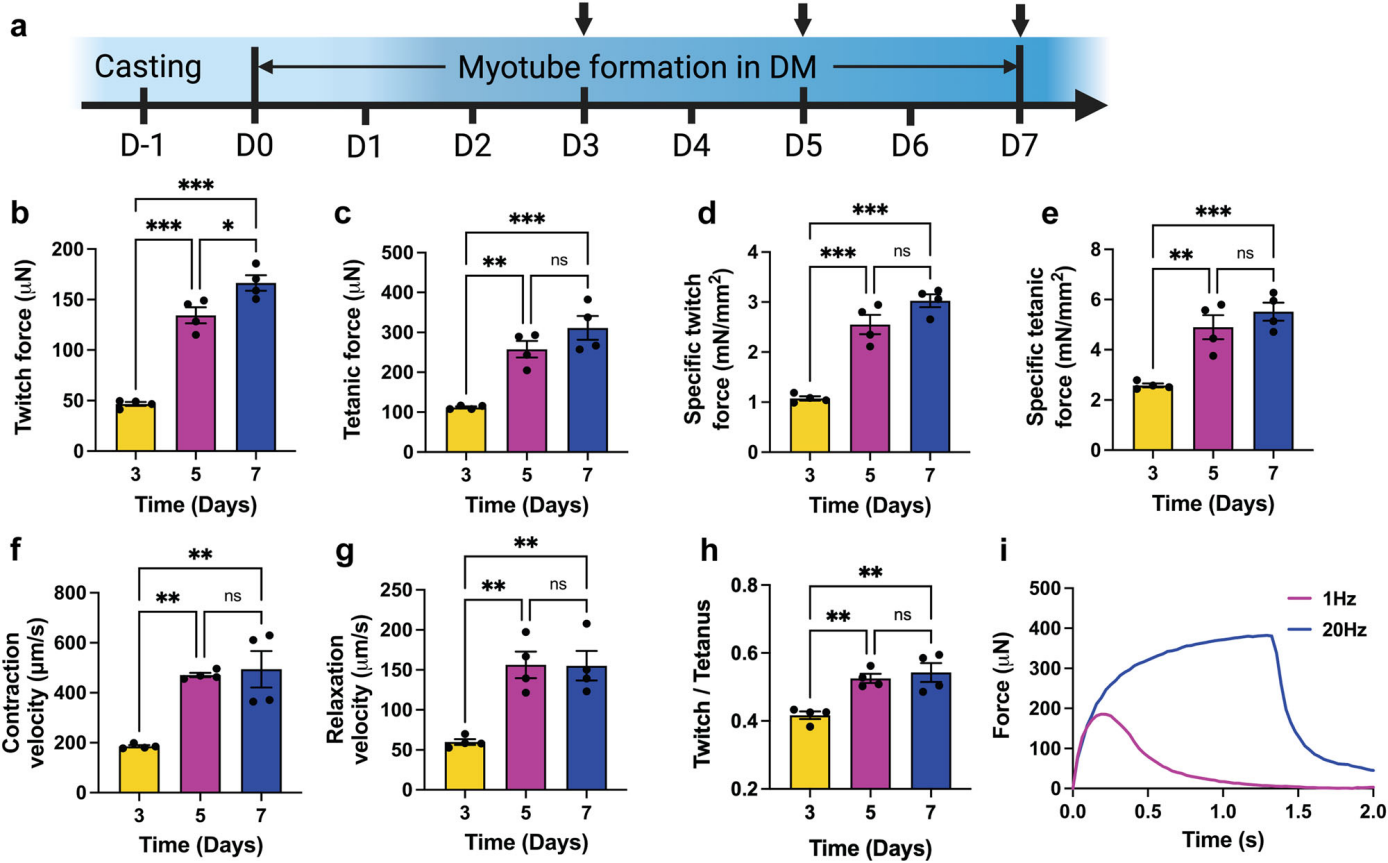
Engineered muscle tissues (EMTs) exhibit improved contractile function in a time-dependent manner. **a** EMT culture procedure. Arrows indicate timepoints for force measurements and immunofluorescence staining. Created using Biorender^®^. **b** Twitch amplitude of EMTs under electrical stimuli (20 V, 1 Hz, 4 ms). **c** Tetanic amplitude of EMTs under electrical stimuli (20 V, 20 Hz, 4 ms). **d** Specific twitch force normalized by total cross-sectional areas of the myotubes. **e** Specific tetanic force normalized by total cross-sectional areas of the myotubes. Contraction of EMTs significantly increases from day 3 (D3) to D5. **f** Twitch contraction velocity of EMTs. **g** Twitch relaxation velocity of EMTs. Significant increase is exhibited in the twitch contraction and relaxation velocities from D3 to D5. **h** Twitch / tetanus ratio significantly increased from D3 to D5. **i** Representative contraction force traces of a 7-day-old EMT show synchronous twitch and tetanic forces as stimulation frequency increases. Data are mean ± SEM (N = 4, ^*^P < 0.05, ^**^P < 0.01, ^***^P < 0.001, ns = no significance by One-way ANOVA followed by multiple comparisons).

To investigate changes in contractile force amplitude of EMTs over time during myogenesis, we determined EMT twitch and tetanic forces in response to electrical stimulation at specific time points (Fig. 2a). EMTs showed synchronous twitch contraction when stimulated with electrical pulses at 1 Hz and exhibited stronger and continuous tetanic contraction when stimulated with electrical pulses at 20 Hz (Fig. 2b, c, i, Supplementary Video 1). Throughout the period of EMT development, twitch and tetanic forces increased significantly by 191.7% and 149.3%, respectively, from day 3 (D3) to D5. Stepwise elevation of twitch and tetanic forces with time indicates myotube formation and improved muscle function following differentiation. While not significantly different from D5, maximal average specific twitch (Fig. 2d, 3.03± 0.26 mN/mm^2^) and tetanic force (Fig. 2e, 5.52 ± 0.72 mN/mm^2^) were reached on D7. Two twitch kinetic parameters, contraction (Fig. 2f) and relaxation velocities (Fig. 2g) showed significant increase from D3 to D5. The twitch / tetanus ratio also significantly increased from D3 to D5 (Fig. 2h). Since twitch force is limited by the incomplete activation and deactivation of microfilaments^27^, an increase of the twitch / tetanus ratio after D3 indicates an accelerated myosin / actin interaction in more developed EMTs at later timepoints. Taken together, these results indicate myotube development in EMTs with time, supporting the use of EMTs as a tool to determine the impact of simulated microgravity on myogenesis in 3D. In addition, significant improvement of EMT contractile function was exhibited before D5, therefore a 5-day myogenic culture period in DM was used in further experiments.

### Simulated microgravity reduces forces, contraction and relaxation velocities of engineered skeletal muscle tissues

**Fig. 3:**
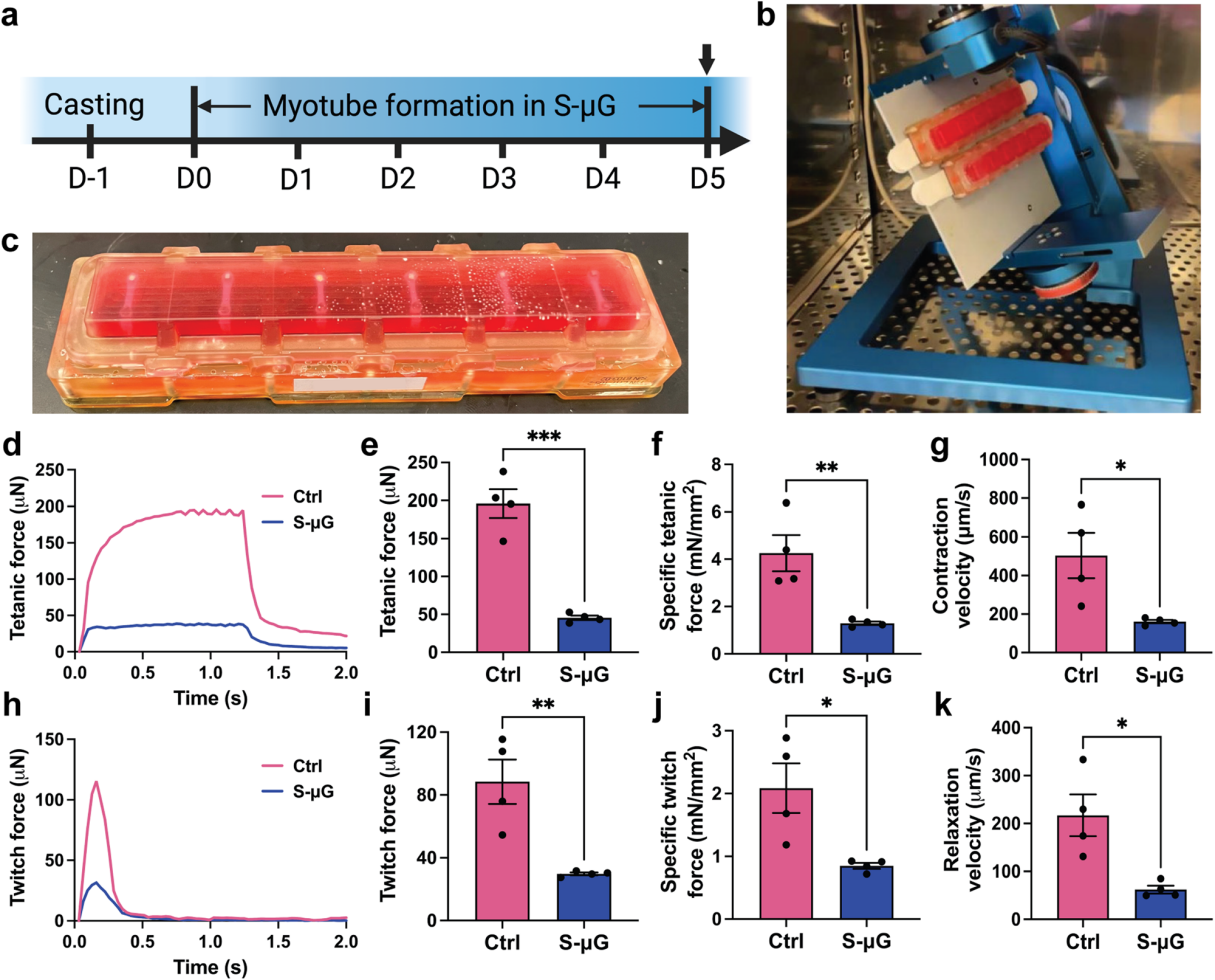
Five-day simulated microgravity (s-μG) reduces contractile function of engineered muscle tissues (EMTs). **a** Experimental timeline. Arrow indicates timepoints for force measurement, immunofluorescence staining, and biochemical analysis of EMTs. Created using Biorender^®^. **b** A random positioning machine (RPM) is utilized to simulate microgravity. **c** A PDMS post array loaded with EMTs is sealed in the tissue chamber for the RPM experiment. **d**, **e** Tetanic amplitude of EMTs under electrical stimuli at 20 Hz and 20 V. **f** Specific tetanic force normalized by total cross-sectional areas of the myotubes. **g** Twitch contraction velocity of EMTs. **h**, **i** Twitch amplitude of EMTs under electrical stimuli at 1 Hz and 20 V. **j** Specific twitch force normalized by total cross-sectional areas of the myotubes. **k** Twitch relaxation velocity of EMTs. Contraction amplitude of EMTs developed in s-μG is significantly lower compared to standard gravity controls (ctrl), accompanied with significantly decreased twitch contraction and relaxation velocities. Data are mean ± SEM (N = 4, ^*^P < 0.05, ^**^P < 0.01, ^***^P < 0.001 by unpaired *t* test).

Once the EMT system was validated, we fabricated tissues to study the effects of reduced gravitational forces on myogenic differentiation in 3D. After a 5-day culture period in sealed tissue chambers while exposed to either simulated microgravity or standard gravity (Fig. 3a, c), EMT physiological function and structure was assessed. To simulate microgravity, EMTs were cultured on the RPM (Fig. 3b) (Supplementary Video 2). First, we focused on synchronous active contraction of EMTs maintained in different gravity conditions. When electrically stimulated, control EMTs that were maintained in standard gravity for 5 days generated twitch (88.42 ± 28.32 μN) and tetanic (195.93 ± 37.68 μN) forces at the same magnitude as the previously presented forces of day 5 EMTs (Figs. 2b, c, 3e, i). This result indicates that 5-day culture in the sealed tissue chamber does not interfere with physiological functions of EMTs. However, EMTs cultured in simulated microgravity for 5 days exhibited significantly reduced tetanic and twitch forces (by 66.3% and 76.8%, respectively, Fig. 3d, e, h, i), accompanied by a significant decrease in specific forces (Fig. 3f, j) comparing to standard gravity controls. Twitch contraction and relaxation velocities of EMTs developed in simulated microgravity were significantly lower than those of control groups (Fig. 3g, k). Taken together, EMTs developed in simulated microgravity demonstrated compromised physiological functions.

### Simulated microgravity reduces myotube length, diameter, and myoblast fusion indices of engineered skeletal muscle tissues

**Fig. 4:**
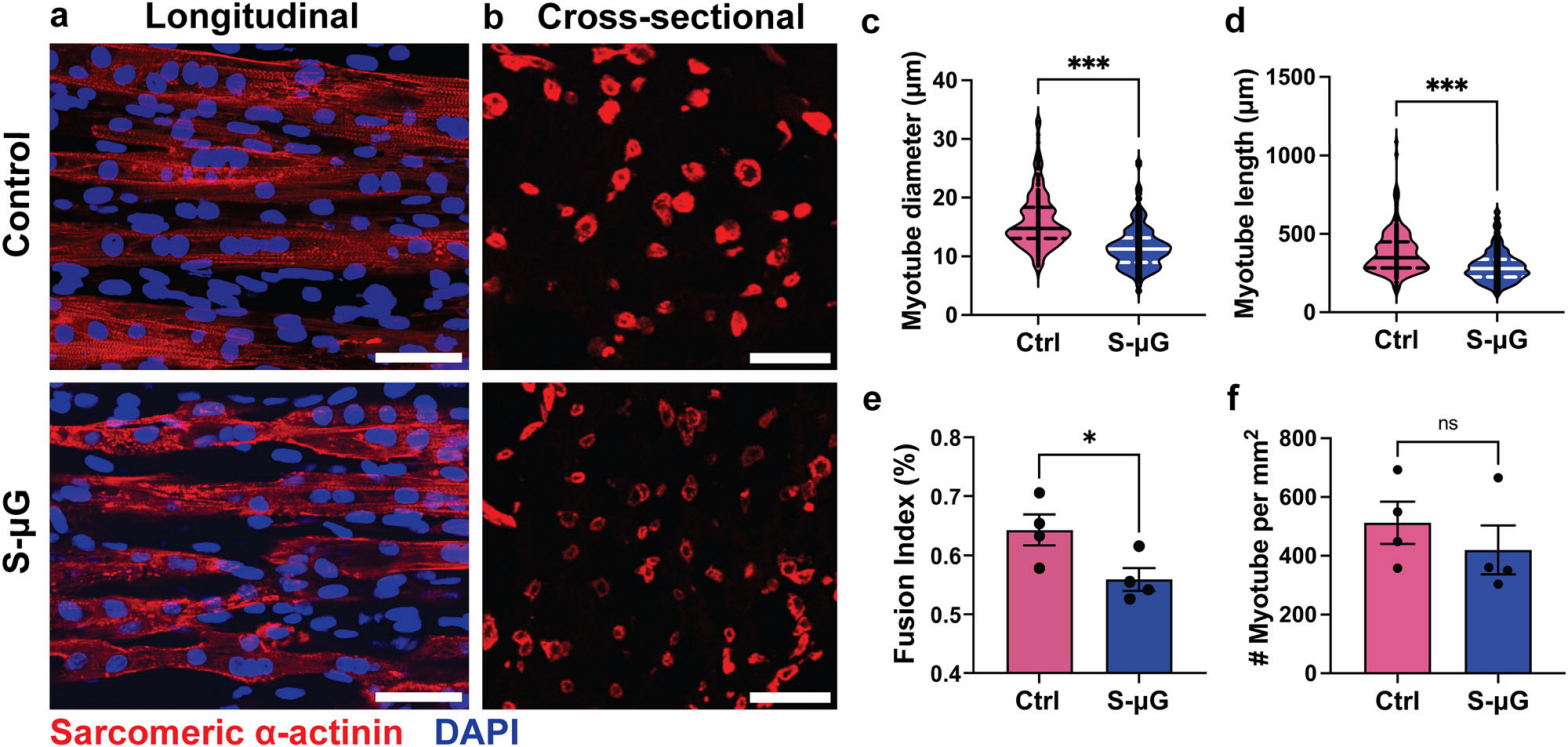
Simulated microgravity (s-μG) decreases myotubular dimensions of engineered muscle tissues (EMTs). **a** Representative image of myotubes in EMTs in standard gravity controls (ctrl) and s-μG. Scale bar: 50 μm. **b** Representative image of EMT cross-sections in controls and s-μG. Scale bar: 50 μm. **c** Average transverse diameter of myotubes. **d** Average length of myotubes. **e** Fusion index of skeletal myoblasts. **f** Number of myotubes in unit cross-sectional area (mm^2^). Diameter, and length of myotubes, and fusion index of myoblasts of EMTs developed in s-μG for 5 days are significantly lower than controls. Myotube density throughout the EMT cross-section shows no statistical significance. Data are mean ± SEM (**c**, **d** N = 200 myofibers, **e**, **f** N = 4 EMTs, ^*^P < 0.05, ^***^P < 0.001, ns = no significance by unpaired *t* test, solid and dotted lines in the violin plots represent means and quartiles respectively).

As myotubes developed in simulated microgravity tend to exhibit reduced contraction, we immunolabeled EMTs for the muscle specific protein sarcomeric α-actinin (SAA) to investigate whether the functional impairment is related to changes in myotubes, which are the contractile structure of skeletal muscle. We first investigated the dimensions of myotubes, including transverse diameter and length. After 5 days of differentiation in either simulated microgravity or standard gravity, myoblasts embedded in 3D hydrogels fused into elongated myotubes as shown in whole-mount and cross-sectional SAA images (Fig. 4a, b). However, myofiber sizes varied with gravity conditions. The transverse diameter of individual myotubes of EMTs maintained in simulated microgravity decreased significantly by 25.8% when compared to controls (Fig. 4c). Length of individual myotubes developed in simulated microgravity also significantly reduced by 23.7% compared to controls (Fig. 4d). These results indicate dimensional deficits of individual myotubes that occurred during the 3D myogenesis in simulated microgravity, which correlate with the observed deficits in functional performance. In addition to structural reduction of individual myotubes, the number of myotubes in each EMT can also directly impact active forces generated by the tissue. Therefore, we analyzed the myoblast fusion indices and myotube density based on SAA-labeled EMT cross-sections. Average myocyte fusion indices showed a significant 12.9% decrease in simulated microgravity groups compared to the controls (Fig. 4e), although there was no statistical significance in myotube density between simulated microgravity and standard gravity groups (Fig. 4f). Taken together, these results indicate that deficits of myotube formation and dimensions correlate with reduction of contractile force.

### Simulated microgravity reduces the expression of proteins related to structures, myogenesis, and mitochondrial biogenesis in engineered skeletal muscle tissues

**Fig. 5:**
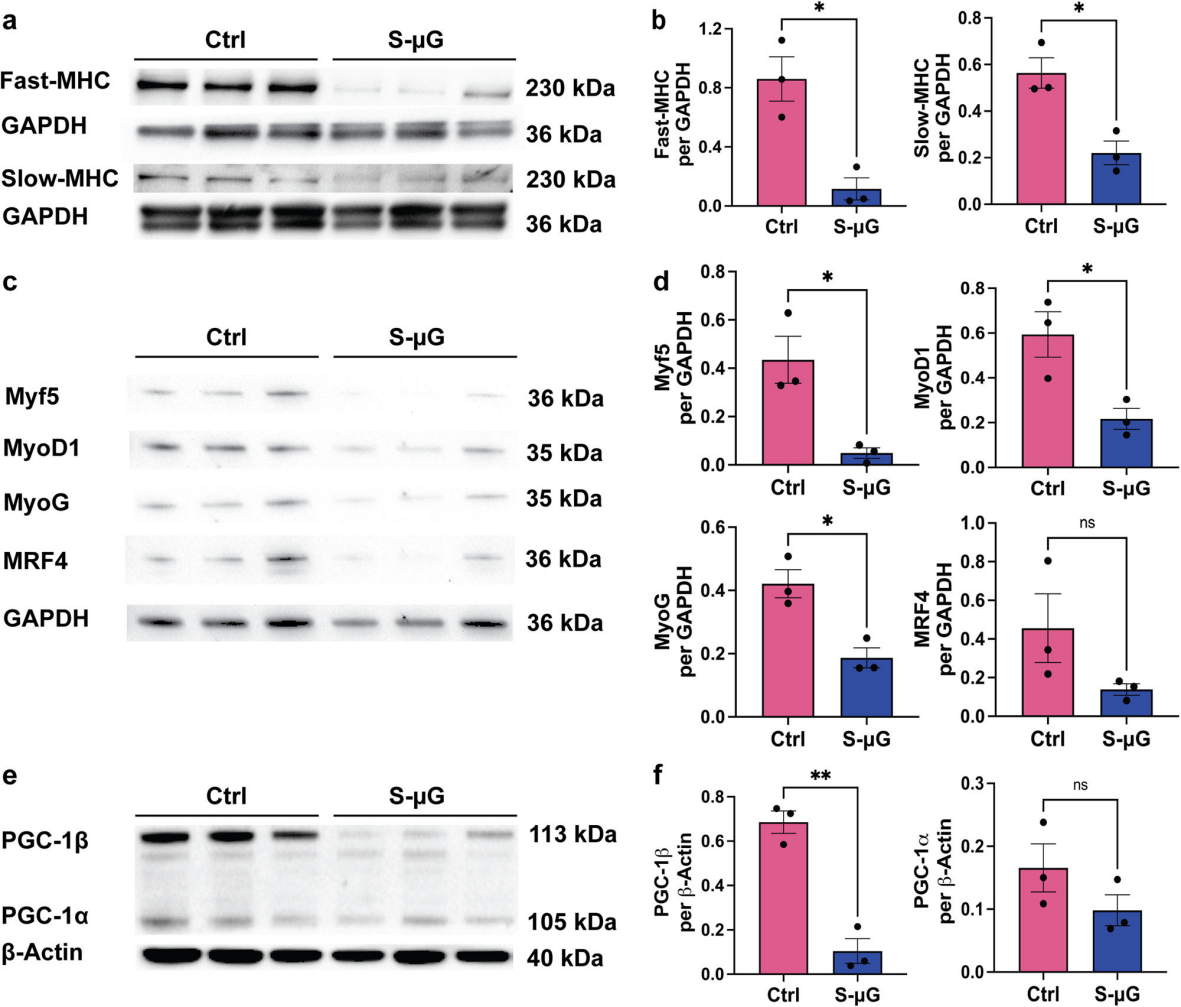
Simulated microgravity (s-μG) reduces the expression of muscle and mitochondrial biogenesis-related proteins in engineered muscle tissues (EMTs). **a**, **b** The expressions of slow- and fast-myosin heavy chain (MHC) proteins are normalized to GAPDH. **c**, **d** The expressions of helix-loop-helix MRF proteins are normalized to GAPDH. **e**, **f** The expressions of peroxisome proliferator-activated receptor (PPAR)-γ coactivator-1α and β (PGC-1α, β) proteins are normalized to β-actin. Expressions of the slow-MHC, fast-MHC, Myf5, MyoD1, MyoG, and PGC-1β are significantly lower after 5-day s-μG compared to normal gravity controls, suggesting compromised myogenic differentiation and mitochondrial function. Data are mean ± SEM (N = 3, ^*^P < 0.05, ^**^P < 0.01, ns = no significance by unpaired *t* test).

To investigate effects of microgravity on myogenesis, we determined the expression of skeletal muscle-specific proteins, fast- and slow- type myosin heavy chains (MHC), which are critical for muscle physiological functions, as well as helix-loop-helix myogenic regulatory factors (MRF), Myf5, MyoD1, myogenin (MyoG) and MRF4, which modulate differentiation of skeletal myoblasts. Immunoblot results demonstrated that the expression of slow- and fast-MHC were significantly reduced to 36.8% and 20.7%, respectively, in simulated microgravity compared to standard gravity control groups (Fig. 5a, b). This finding is consistent with the observed reduction in myotube size. The expressions of Myf5, MyoD1, and MyoG proteins were significantly reduced to 14.3%, 33.6%, and 48.3%, respectively (Fig. 5c, d) in simulated microgravity-groups. Although expression of MRF4 is not statistically significantly reduced in s-μG groups, exhibited a reduced trend. These results support that simulated microgravity inhibit myogenesis in 3D skeletal muscle tissues.

A recent wide-ranging NASA GeneLab data analysis revealed that mitochondrial activity-related genes were significantly altered after spaceflight^12^. A vital mitochondrial biogenesis regulator, peroxisome proliferator-activated receptor (PPAR)-γ coactivator 1 (PGC-1), has also been reported to be related with skeletal muscle myotube development^28–31^. To investigate a correlation between PGC-1 and compromised myogenesis in simulated microgravity, we determined expression of two isoforms of PGC-1, α and β (Fig. 5e, f). Interestingly, expression of PGC-1β protein was significantly downregulated in simulated microgravity groups compared to controls, while expression of PGC-1α protein exhibited a decreasing trend. These results suggest impaired myogenesis related to mature skeletal muscle contractile elements and bioenergetics.

## Discussion

Reductions in contractile force and mass of animal and human skeletal muscle due to microgravity have been extensively reported^5–7^. Direct and indirect factors that mediate this microgravity-induced atrophy include reduction in mechanical loading caused by weightlessness, space radiation, and other unique environmental aspects such as altered nutrition and hormonal disruptions^4^. In concert with musculoskeletal regulation in vivo, compromised myofiber structure and reduced myotube metabolic function have been shown in both real and simulated microgravity using 2D and 3D in vitro models^19,21,22,24,32–34^. However, a comprehensive methodology that studies the dynamics of in vitro 3D myotube formation and underlying regulatory factors in microgravity is lacking, despite recent interest in the use of microgravity for biomanufacturing. Thus, to improve the understanding of the gravitational effects on skeletal muscle tissues, we proposed a tissue-engineered 3D skeletal muscle tissue chip platform as an enabling in vitro model to reproduce development of myotubes in simulated microgravity using the previously described RPM.

Existing methods to study myogenesis and other intracellular dynamics in microgravity have been limited to 2D myocyte monolayers culture systems that lacked the ability to study twitch and tetanic contractile functions of myotubes^32–34^. Our 3D culture system, with non-invasive contractile measurements, addressed some of the limitations of existing gravitational research. Increasing contraction amplitude and dynamics during a 7-day culture demonstrated that myoblasts fused into myotubes possessing intact excitation-contraction coupling and responsiveness to electrical stimuli, while also demonstrating the stability of EMTs over a 7-day period, thus justifying their use in evaluating 3D myoblast differentiation in simulated microgravity. Our 3D cell culture system will benefit gravitational research of the musculoskeletal system. First, the miniaturized dimensions of the EMTs provides a method to study the direct effects of simulated microgravity on skeletal muscle tissue at the cellular level while assessing physiological responsiveness, excluding other confounding factors typical of a complex in vivo environment. EMTs can be further modified into more complex engineered tissues for research involving interactions among various types of cells to study systemic changes in microgravity such as dysregulation of neuromuscular junctions^35,36^. Second, the small scale makes EMTs a promising platform with which experiments can be conducted in a confined operating space, such as the ISS. Compared to animal models, this EMT platform provides an efficient methodology to study mechanisms of microgravity-induced muscle atrophy in spaceflight. While ground-based microgravity-simulation methods cannot fully replicate the gravitational environment of space and may have additional minor factors such as media turbulence and vibration during rotation, they remain a dependable means for conducting preliminary studies prior to actual spaceflight research^20,37^.

During myogenesis, myoblasts exit the cell cycle, followed by morphological changes, cytoskeleton remodeling, and formation of multinucleated myotubes^38,39^. This process was reported to be dysregulated in the microgravity environment^21,22^. Existing studies on myogenesis in microgravity, however, were mostly based on 2D culture systems and failed to address the impact of 3D intercellular crosstalk during myogenesis. Therefore, to study the effects of simulated microgravity on myogenesis in 3D, we cultured EMTs in DM on both the RPM and in standard gravity for 5 days after fabrication. EMTs that were developed in simulated microgravity exhibited attenuated physiological responses compared to standard gravity control groups (Fig. 3). Whole-mount staining results demonstrated that EMTs developed in simulated microgravity possessed myotubes with reduced dimensions and myoblast fusion indices (Fig. 4). In addition, biochemical analysis results showed reduced expression of skeletal muscle-specific MHC proteins (Fig. 5a, b). These results indicate that simulated microgravity attenuates physiological structure and cytoskeletal protein expression of skeletal myotubes in 3D.

We noticed three of the MRFs (Myf5, MyoD1, and MyoG) were significantly reduced in the simulated microgravity groups compared to the controls after 5-days of culture on the RPM (Fig. 5c, d). During myogenesis, expression of Myf5 and MyoD1 are first upregulated in the functional skeletal myoblasts. MyoG and MRF4 will then be upregulated in the initiation of the myogenic differentiation^40^. Reduction of the MRFs indicated that short-term simulated microgravity culture can significantly retard myogenesis. This result is consistent with the previous published works that studied myogenesis of 2D culture systems in short-term simulated microgravity^21,34^. A recent publication that studies the changes of native mouse skeletal muscles in reduced gravity levels showed that after 1-month exposure to microgravity, muscle mass and fiber dimensions were significantly decreased, while MyoD1, MyoG, and MRF4 genes were significantly upregulated^11^. This indicated that during long-term exposure to microgravity, native skeletal muscle tissues can potentially compensate for the muscle loss by reactivating the myogenic pathways.

PGC-1 is highly expressed in tissues with high energy demands, including slow-twitch muscle, cardiac muscle, and adipose tissues^41–43^, and broadly regulates oxidative metabolism, including oxidative phosphorylation and fatty acid oxidation^44^, a key metabolic process for skeletal muscle^45^. PGC-1α, the most characterized member of the PGC-1 family, is responsible for energy homeostasis ubiquitously through other mitochondrial regulators^46,47^. PGC-1β, another isoform of PGC-1, was identified by its highly similar distribution pattern and role in mitochondrial regulation as PGC-1α^28,41^. It is reported that overexpression of PGC-1β partially activates PGC-1α target genes^41,48^. Considering the high metabolic demands of skeletal muscle, the regulation of mitochondrial oxidative metabolism by PGC-1 significantly impacts muscle function. It has been reported that in skeletal muscle, PGC-1α and β play roles in modulating mitochondrial biogenesis^30^ and myosin heavy chain isoforms^28,49^. PGC-1β is reported to be upregulated in differentiating murine myoblasts and to induce mitochondrial biogenesis^29^. Overexpression of PGC-1α/β in murine myotubes resulted in increased tube sizes and protein synthesis^50^. Therefore, we studied the relationship between microgravity-attenuated 3D myogenesis with PGC-1. We observed a significant decrease (by 6.8-fold) in PGC-1β expression at the protein level (Fig. 5e, f). It has been reported that MyoD1 can directly regulate the transcription of PGC-1β in skeletal myocytes^51^. During C2C12 myogenesis, increased binding between MyoD and multiple sites of *PGC-1β* gene was observed, accompanied with the elevated expression of PGC-1β. These results indicated that the compromised myogenesis in microgravity is potentially regulated by the interaction between MRFs and mitochondrial regulators.

In conclusion, we proposed a tissue-engineered 3D skeletal muscle chip to study myogenesis and contractile function using a ground-based microgravity simulation platform. This study applying an in vitro 3D skeletal muscle model that allows non-invasive functional assessment of engineered skeletal muscles demonstrated attenuating effects of simulated microgravity on myotube formation in 3D, as shown by reductions in contractile amplitude, myofiber dimensions, and myogenesis-specific protein expression. It is known that after space travel, skeletal myocytes exhibit force regression and mass loss in a similar manner as aging; therefore, microgravity-exposed skeletal muscles might be a potential model for anti-aging studies for terrestrial benefits. The results presented here provid insights into molecular rationales for changes in myogenesis in simulated microgravity, which can be potentially applied to the mechanisms of age-related muscle dysfunction, the development of an accelerated in vitro aging model, and the evaluation of anti-regression pharmaceuticals.

## Methods

### Fabrication of posts and tissue molds

The customized post pairs consist of a flexible and a rigid post with caps on the tips and were fabricated using polydimethylsiloxane (PDMS, Sylgard 184; Dow Corning, Midland, MI) and custom acrylic molds (Supplementary Fig. 1a) following a published protocol^52^. Briefly, 10 mL of PDMS and the curing agent (10:1 w/w) were homogenized, degassed, and poured into the mold. Glass capillaries tubes (Drummond Scientific Co., Broomall, PA) were then inserted into the opposite posts to make them rigid. After being baked at 65 °C for 17 hours and peeled from the mold, PDMS posts with a height of 12.5 mm (with 0.5 mm caps) and a cross-section diameter of 1.5 mm had been fabricated (Fig. 1a, b).

Customized tissue fabrication molds were manufactured in a commercially available polystyrene 24-well plate (CELLSTAR, Greiner Bio-One, Kremsmünster, Australia). 1.5 mL of PDMS mixed with curing agent (10:1 w/w) was added to each well. Custom 3D-printed inserts were placed into wells to make rectangular fabrication molds in the PDMS. After 17 hours of baking at 65 °C, inserts were removed to generate tissue fabrication wells with a length of 12 mm and a width of 4 mm (Supplementary Fig. 1b, c). EMTs were then fabricated in these customized 24-well plates (Fig. 1c–e).

### Cell culture and maintenance

C2C12 murine skeletal myoblasts (ATCC, cat. CRL-1772) were maintained in growth media (GM) consisting of Dulbecco’s Modified Eagle Medium (DMEM, high glucose, w/ pyruvate; Thermo Fisher, cat. 11995065), fetal bovine serum (FBS, 10% v/v; Thermo Fisher, cat. 26140079), and Penicillin/Streptomycin (1% v/v; Thermo Fisher, cat. 15140122). Myoblasts were cultured in polystyrene flasks with media exchanges every other day and passaged using 0.25% trypsin-EDTA (Thermo Fisher, cat. 25200056) after reaching 70% confluence (about every 2 days). When passaging, myoblasts were seeded at 5,000 cells/cm^2^, following the vendor’s instruction.

### Fabrication and maintenance of engineered muscle tissues

Tissue fabrication molds were immersed in 70% ethanol overnight and PDMS posts were exposed to ultraviolet radiation for 30 min for sterilization before tissue fabrication. PDMS posts were then pre-treated in 0.1% (v/v) poly(ethyleneimine) (PEI, Sigma, cat. 408727) solution for 10 min and 0.01% (v/v) L-glutaraldehyde solution for 30 min in succession. Posts were washed in deionized (DI) water for 5 min after each step, then dried aseptically after pretreatment. Tissue fabrication wells were pre-treated with 5% (w/v) Pluronic F-127 (Sigma, cat. P2443) solution for 30 min followed by a quick rinse with DI water, aseptically dried, then stored at 4 °C until tissue fabrication.

EMTs were formed from a collagen / Matrigel composite hydrogel and were fabricated following a similar protocol as previously described^25^. 500,000 myoblasts suspended in 60 μL of GM were mixed in pre-treated wells placed on ice with 91 μL of rat tail collagen type I (3 mg/mL; Thermo Fisher, cat. A1048301) and 29 μL of ECM gel from Engelbreth-Holm-Swarm murine sarcoma (Sigma, cat. E1270), then neutralized with 0.1 M NaOH solution, until the mixture turned pink. Fabrication wells were transferred to 37 °C, 5% CO_2_ after PDMS post arrays were placed. After 1 h, 1 mL of GM was added to each well and then incubated overnight (17 h) to allow spontaneous compaction of the cell-loaded hydrogel.

### Myotube formation in 3D hydrogel

To induce myotube formation, each EMT was cultured in 1 mL of differentiation media (DM) consisting of DMEM, horse serum (HS, 2% v/v; Thermo Fisher, cat. 16050122), insulin-transferring-selenium-ethanolamine (ITS-X, 1% v/v; Thermo Fisher, cat. 51500056) and Penicillin/Streptomycin for 7 days post-fabrication. DM was changed every day. During this period, EMT samples were utilized at specific time points (Fig. 2a) for contractile function analysis before being harvested for structural analysis at the end of the experiment.

### Microgravity simulation using random positioning machine

An effective system for microgravity environment simulation on the ground, called a random positioning machine (RPM^SW^ 2.0; Yuri Gravity, Meckenbeuren, Germany), was used in this study. The mode of operation of RPM^SW^ 2.0 was previously tested and used to study the effects of simulated microgravity on in vitro osteoblast function^53^. The RPM^SW^ 2.0 can rotate the samples at a conventional random motion to generate a microgravity (as low as 10^-3^G) on the sample stage, which was controlled by pre-determined path file “p0b.txt” developed and validated by the manufacturer (Supplementary Fig. 3).

Twenty-four EMTs were transferred to four sealed custom tissue culture chambers out of PDMS-PEG (Fig. 3c; BioServe Space Technologies, University of Colorado, Boulder, CO) filled with DM after fabrication and overnight culture. These chambers are constructed with a 1% PDMS-PEG block copolymer membrane to prevent absorption of differentiation factors by PDMS as previously described^56^. After carefully removing air bubbles, two chambers, each containing 6 EMTs, were fixed to the center of the sample stage on RPM (Fig. 3b) and rotated while two chambers were incubated statically serving as control groups. All four chambers and the RPM were incubated at 37 °C, 5% CO_2_ for 5 days, then harvested for further analysis.

### Electrical stimulation experiments and contractile analysis

Field electrical stimulation was applied to EMTs to test their contractile function. Copper electrodes were affixed onto a 3D printed support, which placed pairs of anodes and cathodes on both sides of EMTs with a 1 cm distance in between (Supplementary Fig. 1d-e). Before stimulation, EMTs cultured for specific time periods were transferred to a stimulation chamber filled with pre-warmed, fresh DM with electrodes immersed inside. Electrodes were then connected to a MyoPacer (IonOptix, Westwood, MA). The electrical stimulation chamber was then incubated at 37 °C, 5% CO_2_.

Field electrical stimulation with the voltage of 20 V and pulse width of 4 ms were generated between electrode pairs by the MyoPacer. EMTs were stimulated at different frequencies (1 Hz and 20 Hz), to measure twitch and tetanic forces, respectively. Contractile forces were analyzed using an image-based method. Deflection of the flexible post caused by EMT contraction was recorded by a camera at 30 fps and tracked using a customized MATLAB (MathWorks Inc., Natick, MA) code (Supplementary Fig. 1f). Forces were calculated based on Young’s modulus of the flexible posts and the tracked deflection using Euler-Bernoulli beam theory. Twitch contraction and relaxation velocities were calculated from twitch force, contraction, and relaxation time.

### Sample collection for structural and biochemical assessment

To avoid additional influential factors, EMTs were only electrically stimulated once at expected timepoints, then harvested for further structural or biochemical analysis. For immunofluorescence staining, EMTs were carefully removed from the PDMS posts, washed with DPBS, then fixed in 4% paraformaldehyde (PFA) in PBS overnight at 4 °C, then transferred to DPBS and stored no longer than 7 days before staining and observing. For immunoblotting, protein samples were collected using RIPA lysis and extraction buffer (Sigma, cat. R0278) with Protease/Phosphatase Inhibitor (Cell Signaling, cat. 5872 S), after thoroughly grinding the EMTs using a Dounce tissue grinder (VWR, cat. 62400-595). After centrifuging the tissue extractions, supernatants were collected and preserved in −80 °C before immunoblot analysis.

### Immunofluorescence staining

Brightfield images were captured using a Nikon ECLIPSE Ti inverted microscope. Whole-mount immunofluorescence staining was conducted for histological analysis. After fixation, samples were washed three times in DPBS, 5 min each time, followed by 20 min permeabilization in 0.3% Triton-X 100 and 2 h blocking in 1% (w/v) bovine serum albumin (BSA) with 0.1% (v/v) Tween 20. For sarcomeric alpha-actinin (SAA) staining, rabbit anti-SAA antibody (Abcam, cat. ab68167) was applied overnight at 4 °C and anti-rabbit IgG Alexa Fluor 594 (Abcam, cat. ab150080) was subsequently applied at room temperature for 2 h.

Cross-sectional images were acquired for cross-sectional area and specific force assessment. After fixation, EMT samples were dehydrated using 30% (w/v) sucrose solution for 30 min, dried on Kimwipes^®^ (Kimtech Science, cat. 06-666), then embedded in Scigen Tissue-Plus^TM^ OCT compound (Fisher, cat. 23-730-571) and frozen in dry ice overnight. Embedded EMT samples were then cross-sectioned into 4-μm cryosections near the center, permeabilized in 0.3% Triton-X 100 for 3 min, blocked in 1% (w/v) BSA with 0.1% (v/v) Tween 20, then stained for SAA following the above-mentioned procedure. Images were acquired using Leica SP8 inverted confocal microscope and LAS X image processing software.

### Myotube dimension, density, and myoblast fusion index analysis

Myotube sizes, density and myoblast fusion indices were measured by assessing 10X and 20X magnification confocal images of EMTs and cryosections after immunofluorescence staining for SAA and DAPI. For myotube size assessment, z-stack images were taken near the center of EMTs and then analyzed to quantify the length of each myotube using ImageJ version 2.3.0^54^. Lengths and areas of individual myotubes were assessed using longitudinal staining of the EMTs. The average diameters of the myotubes were subsequently determined by dividing their areas by their lengths. A minimum of 50 myotube lengths and diameters were analyzed per EMT. Myoblast fusion indices were calculated by dividing the number of myotube cell nuclei vs the total number of nuclei. Myotube density was calculated as number of myotube per mm^2^ of EMT cross-sections and analyzed using ImageJ.

### Immunoblot analysis

The concentration of total proteins was quantified using the Pierce BCA assay kit (23225, Thermo Fisher). To determine expressions of MHC, Myf5, MyoD1, MyoG, MRF4, and GAPH proteins, immunoblot analyses were performed as described previously^55^ with minor modifications. Proteins were fractionated on NuPAGE 4-12% Bis-Tris protein gels (NP0335BOX, Thermo Fisher) via XCell SureLock Blot Module (EI0002, ThermoFisher) and transferred onto nitrocellulose or PDVF membranes. Primary antibodies used were: fast-MHC (Abcam, cat. ab91506), slow-MHC (Abcam, cat. ab228353), MyoD1 (Abcam, cat. ab203383), MyoG (Abcam, cat. ab124800), Myf5 (R&D Systems, cat. AF4027), MRF4 (Abclonal, cat. A15291), PGC-1α, β (Abcam, cat. ab54481), β-actin (Sigma-Aldrich, cat. A1978), and GAPDH (Cell Signal, cat. 5174 S). The blots were detected with Amersham ECL select western blotting detection reagent (Millipore Sigma) or SuperSignal West Pico Plus chemiluminescence substrate (Pierce, ThermoFischer) and photographed using a digitalized ChemiDoc XRS+ system (Bio-Rad Laboratories, Hercules, CA). The intensity of each protein band was quantified using Adobe Photoshop 2021 (Adobe Inc.) and the values were normalized to the intensity of endogenous proteins β-actin (for PGC1α, β) or GAPDH (for slow-, fast-MHC, MyoD1, MyoG, Myf5, and MRF4).

### Statistical analysis

All experiments were repeated three times individually to confirm the reproducibility. All numeric data was processed using Microsoft Excel 2013 (Redmond, Washington). Images were quantitatively analyzed using ImageJ. Image-based tissue contraction analysis was conducted using MATLAB R2022a version 9.12.0 (MathWorks Inc., Natick, Massachusetts). Values were analyzed and plotted using GraphPad Prism version 9.3.1 (GraphPad Software, San Diego, California) and graphs were presented as mean ± standard error of the mean (SEM). Data normality was analyzed using the Shapiro-Wilk test. Statistical significance was analyzed by unpaired *t* test or one-way ANOVA with Tukey’s multiple comparisons test. Two-sided *P*-values are annotated as ^*^P < 0.05, ^**^P < 0.01, and ^***^P < 0.001.

### Supplementary information

Active tetanic contraction of engineered skeletal muscle tissues (EMTs) in response to electrical stimuli.

Active twitch contraction of engineered skeletal muscle tissues (EMTs) in response to electrical stimuli.

Engineered skeletal muscle tissues (EMTs) sealed in tissue chambers were maintained on a random positioning machine (RPM).

Supporting Information

## Data Availability

The data that support the findings of this study are available from the corresponding authors upon reasonable request.
