# Supplementary material for: Simulated microgravity attenuates myogenesis and contractile function of 3D engineered skeletal muscle tissues
Source: NPJ Microgravity. 2024 Feb 16;10:18. doi: 10.1038/s41526-024-00353-z (PMC10873406; doi:10.1038/s41526-024-00353-z)
Supplement: Supplementary file 4 — Supporting Information [file 41526_2024_353_MOESM4_ESM.docx]

Supporting Information

**Simulated Microgravity Attenuates Myogenesis and Contractile Function of 3D Engineered Skeletal Muscle Tissues**

*Zhanping Ren, Eun Hyun Ahn, Minjae Do, Devin B. Mair, Amir Monemianesfahani, Peter H.U. Lee^#^,*

*and Deok-Ho Kim^#^*


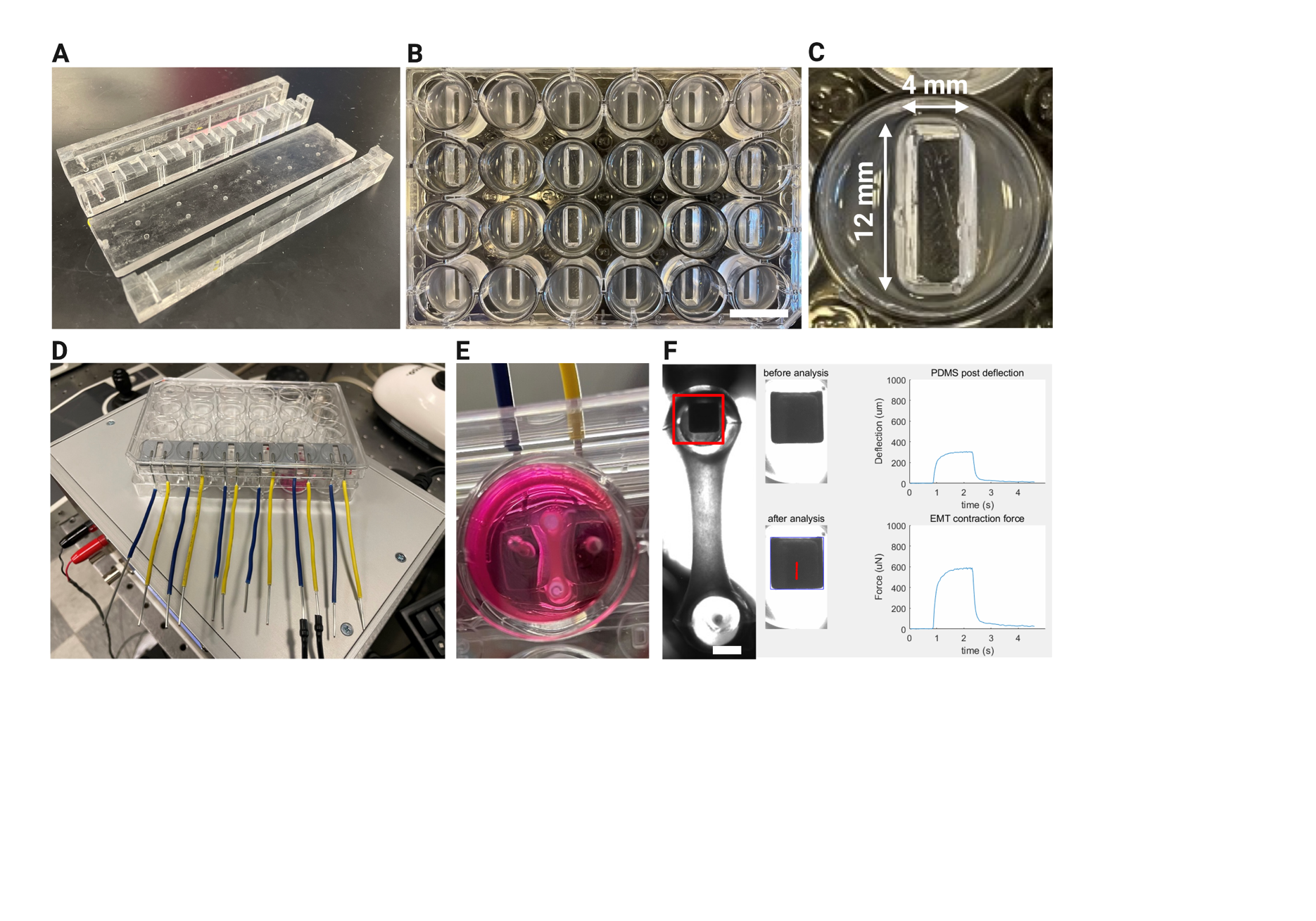
*^#^: Authors for correspondence*

**f**

**e**

**d**

**c**

**b**

**a**

**Supplementary Figure 1. PDMS post mold, tissue fabrication wells, and electrical stimulation device.** (**a)** Acrylic mold for PDMS post fabrication. (**b)** Tissue fabrication wells were fabricated using PDMS on a standard 24-well plate. Scale bar: 15 mm. (**c)** Zoomed-in image of a fabrication well with a length of 12 mm and a width of 4mm. (**d)** Customed electrical stimulation device for engineered muscle tissues (EMTs). Electrodes are connected to commercial IonOptix MyoPacers. **(e)** Zoom-in image of electrode pairs placed on both sides of an EMT. Field stimulation generated by electrodes induce contraction of EMTs. (**f)** Movement of flexible posts (red square) due to EMT contraction was tracked optically based on which forces were calculated using customized MATLAB code. Scale bar: 1 mm.


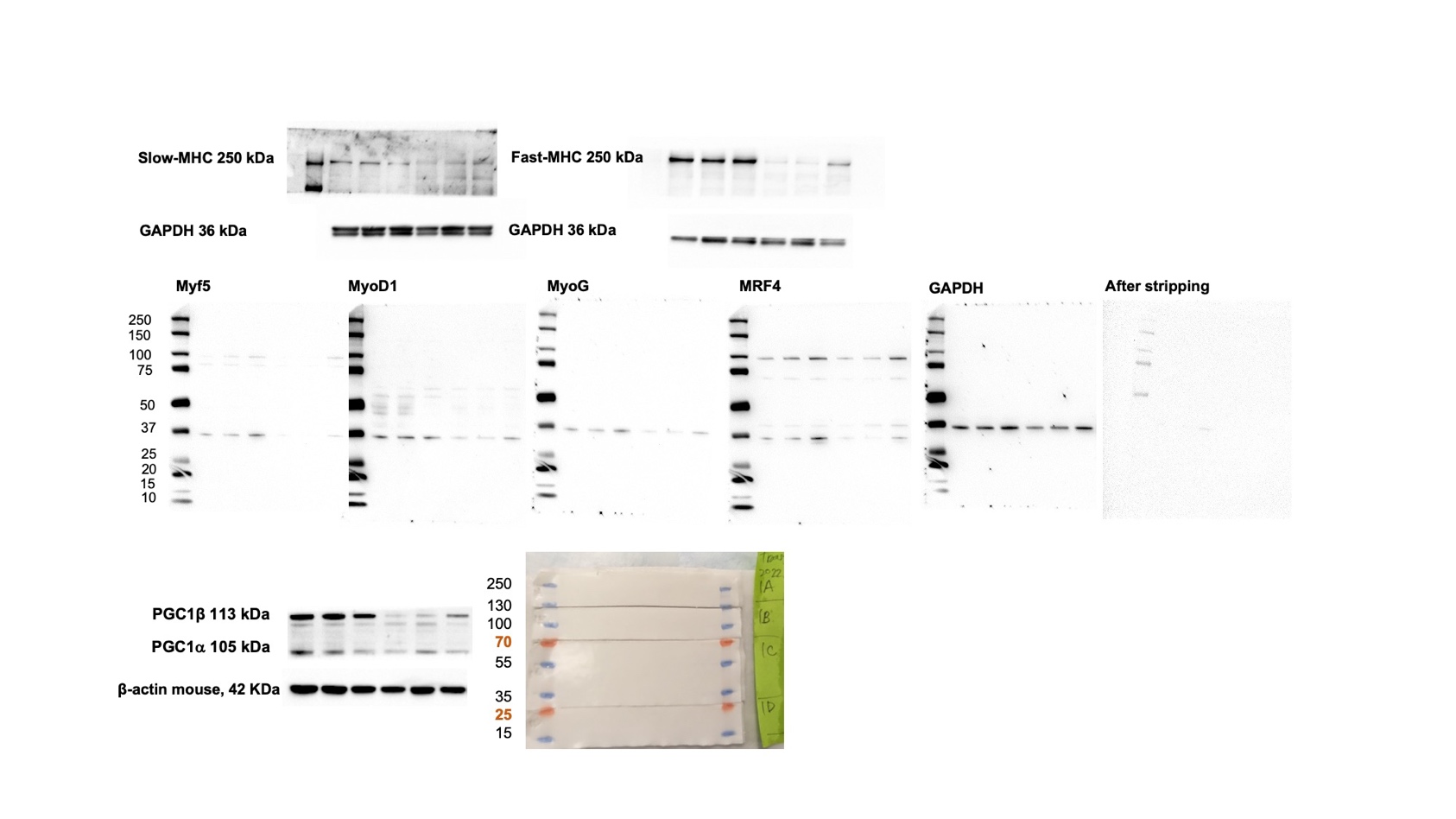


**Supplementary Figure 2. Full-membrane image of the immunoblotting data.** Molecular weight (kDa) is labeled on the left.


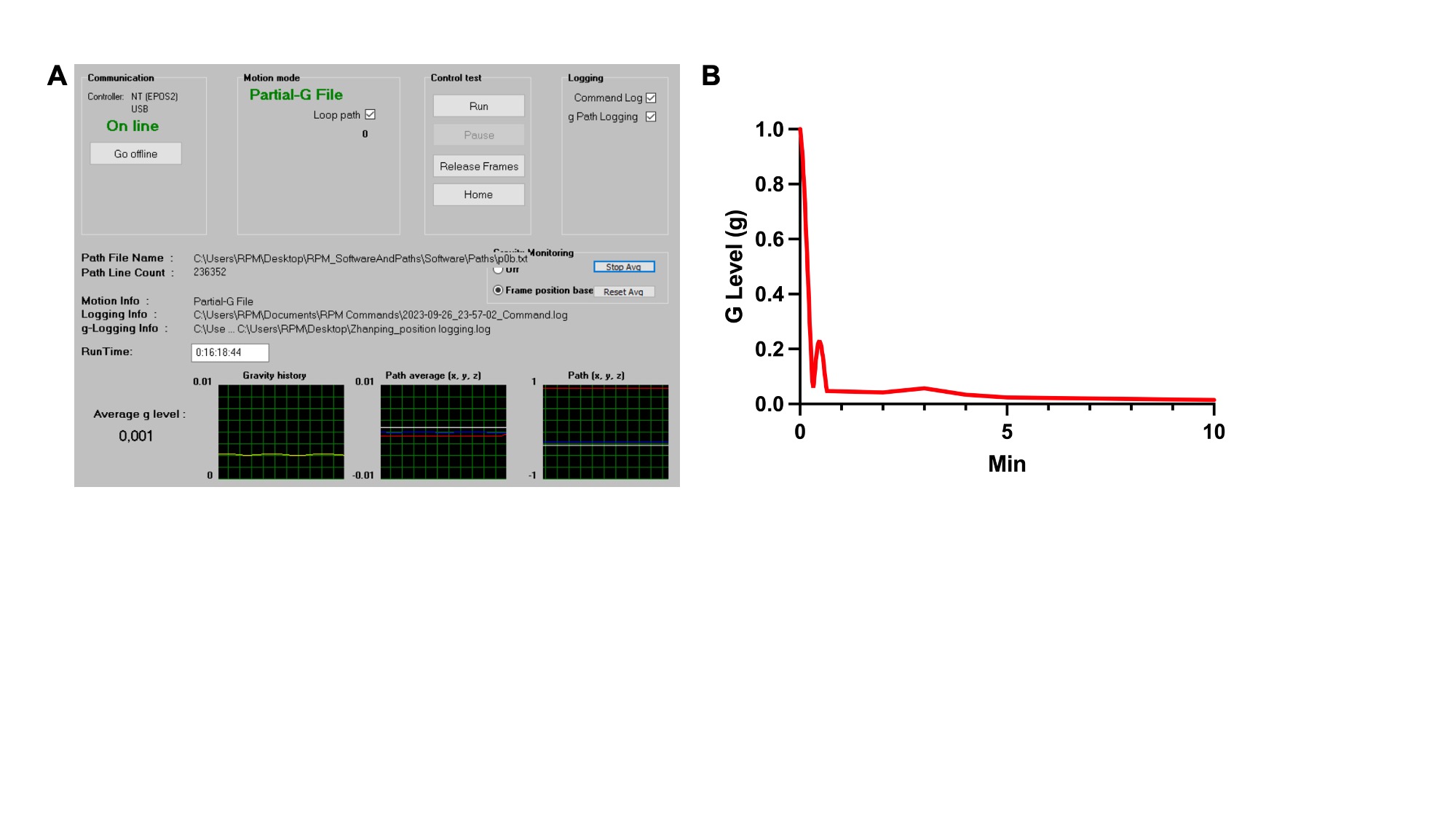


**b**

**a**

**Supplementary Figure 3. Motion mode of the random positioning machine. (a)** RPM^SW^ 2.0 control panel allows selection of the path-file provided by the manufacturer, tracking of the average G level and position. Controlled by the path-file p0b.txt, the average G level on the sample stage can be stabilized at 10^-3^ G in 1.5 hrs. **(b)** Gravity (G) level drastically dropped within 1 min after the RPM^SW^ 2.0 was initiated, then stabilized after 5 min. G level reached and remained at 10^-3^ G after 90 min (datapoints not shown).


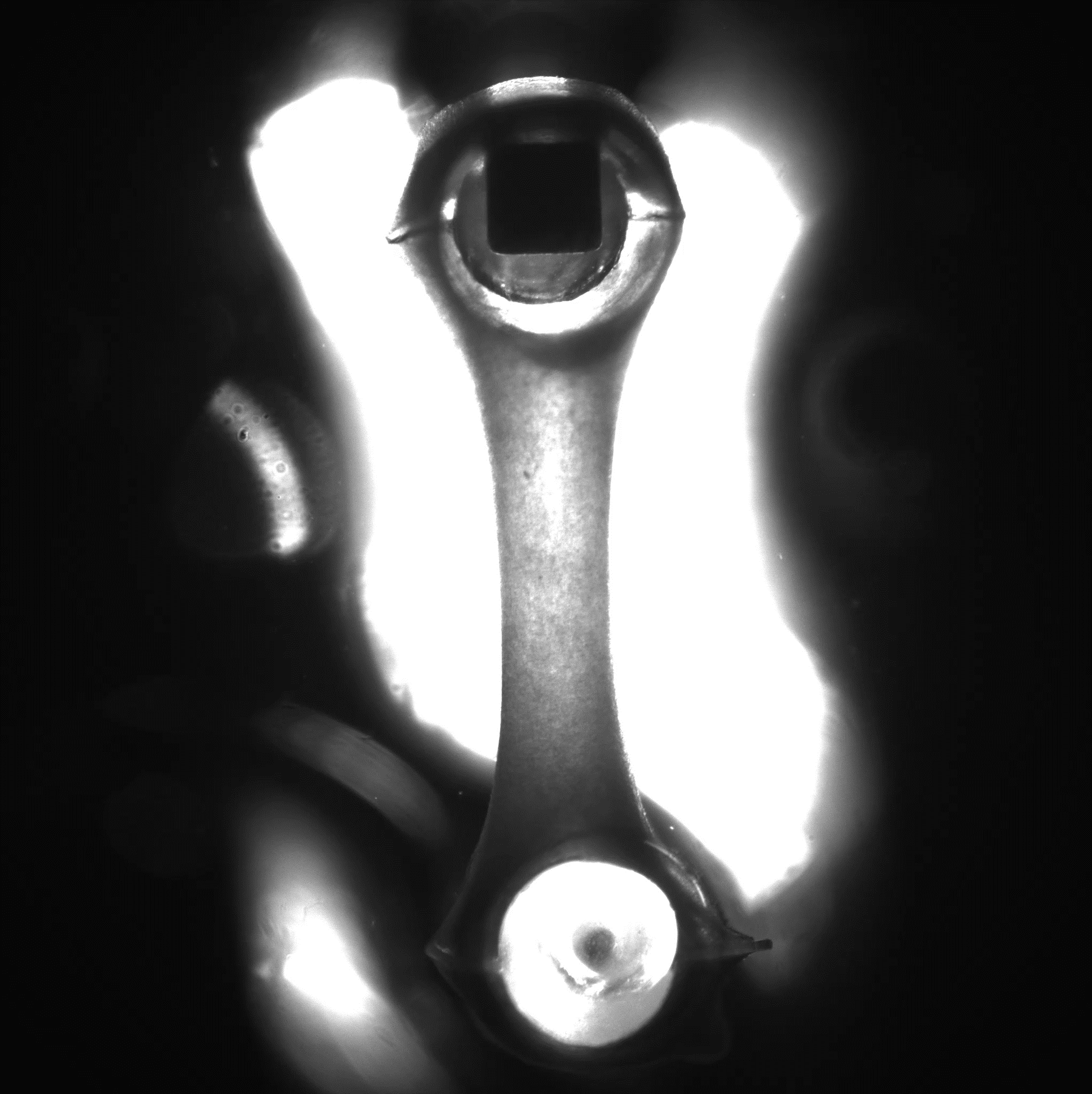

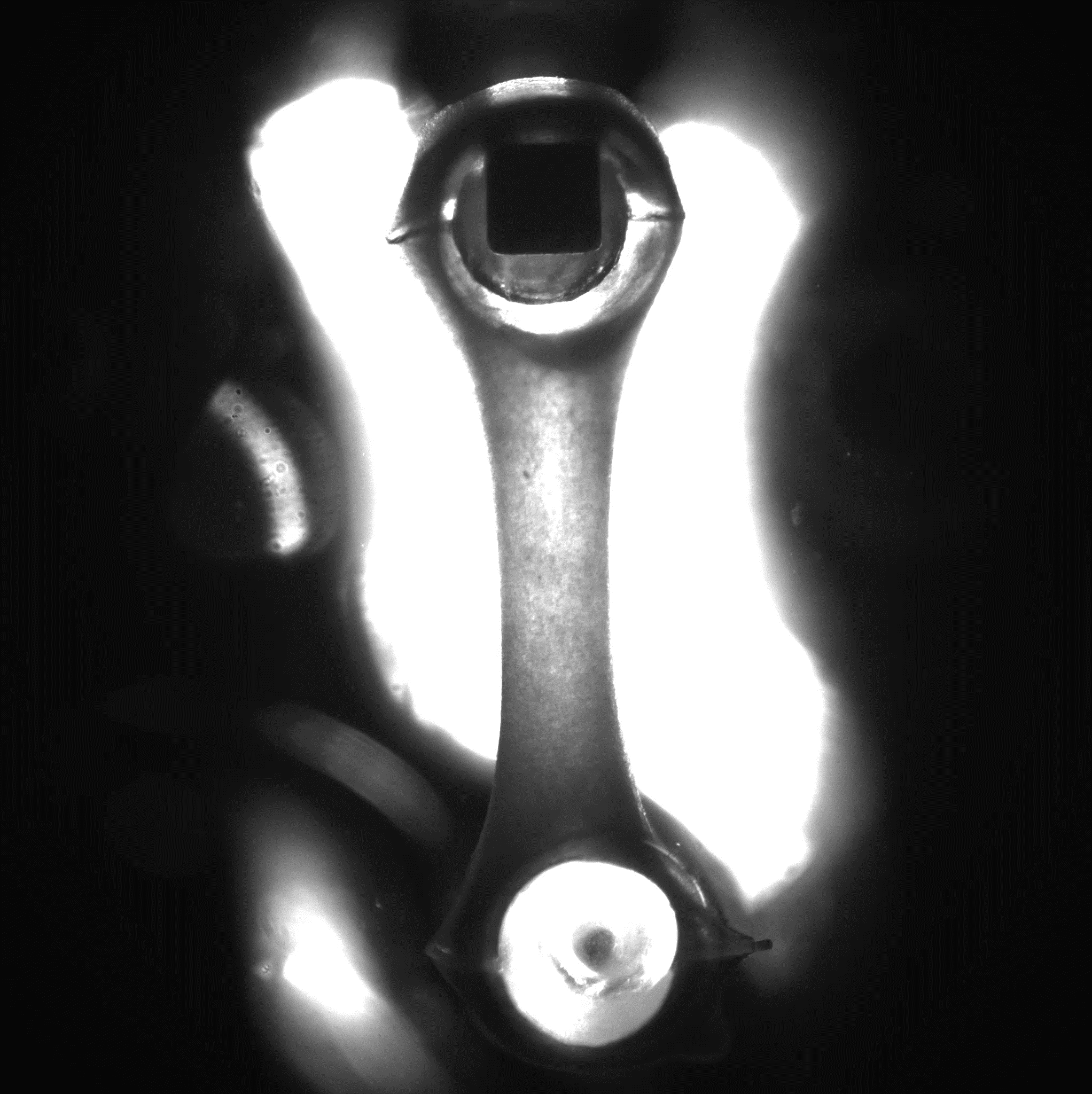


**Supplementary Video 1. Active twitch and tetanic contraction of engineered skeletal muscle tissues (EMTs) in response to electrical stimuli.**

Tetanus

Twitch


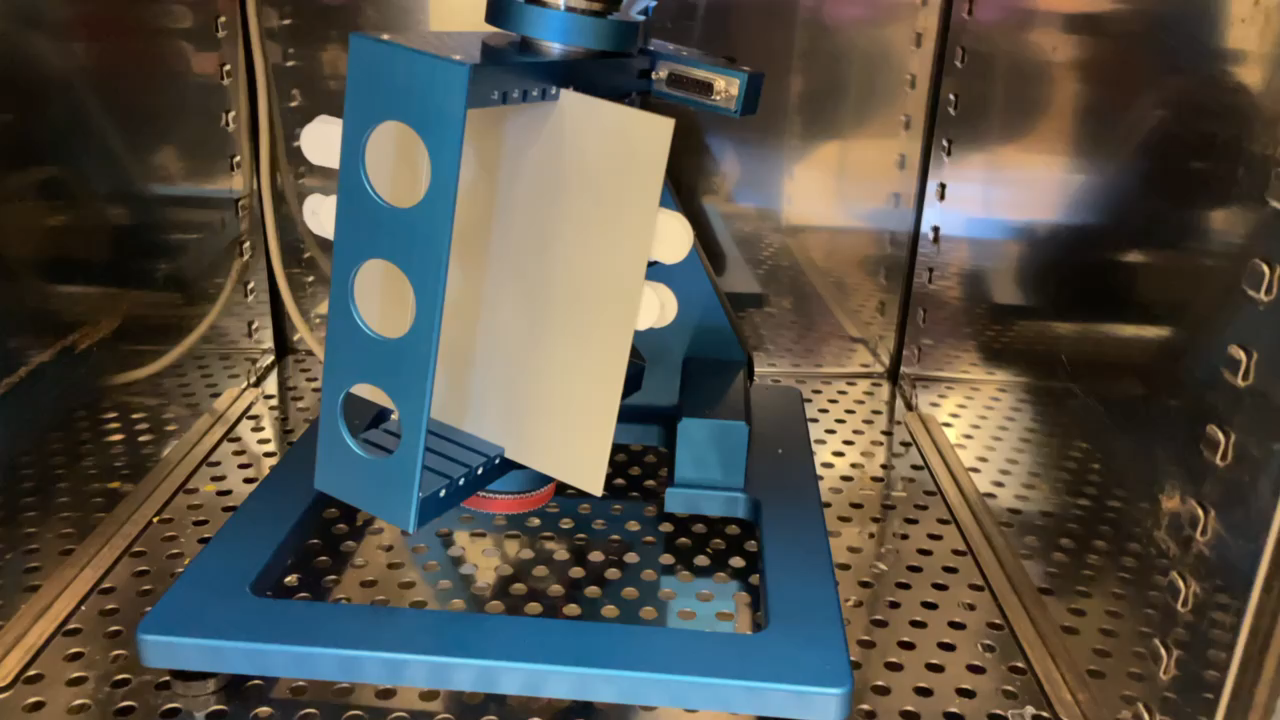


**Supplementary Video 2. Engineered skeletal muscle tissues (EMTs) sealed in tissue chambers were maintained on a random positioning machine (RPM).**
